# Social movements and social media: the evolution of scholarship in the age of datafication

**DOI:** 10.1057/s41599-026-07128-9

**Published:** 2026-04-28

**Authors:** Aya Shoshan, Jennifer Oser, Francisca Castro

**Affiliations:** 1 Ben-Gurion University of the Negev, Beersheba, Israel; 2 Carlos III University of Madrid, Madrid, Spain

**Keywords:** Cultural and media studies, Politics and international relations, Science, technology and society, Sociology

## Abstract

The evolution of social movements in the age of datafication has challenged prevailing social movement theories. Contrary to scholarly forecasts, datafication empowered social movements by reducing the cost of participation and removing barriers to disseminating information, while also facilitating authoritarianism and imposing new constraints on movements’ organizational dynamics and long-term impact. While literature reviews of the field suggest that these gaps have largely been overcome, little is known about how the field as a whole has evolved to address these questions. Using bibliometric tools to visualize and analyze a dataset of 6701 studies on social media and social movements published between 2005 and 2023, we identify the canonical literature, research approaches, and research methods used to study these developments. Our findings highlight a consistent scarcity in experimental research that isolates causal mechanisms, and we point to experimental work in related fields that could advance this line of research. We conclude by discussing opportunities for next-step research that emerge from our findings, including the use of big data analysis as well as qualitative and experimental studies to address unresolved questions about social movements in the age of datafication.

## Introduction

Two major developments occurred concurrently in the late 2000s: a few years after several major social media platforms launched, mass protests erupted across the globe, including the Arab Spring uprisings and Occupy Wall Street. While scholars have questioned a causal relationship between the two (Fuchs, 2024; Fuchs and Sandoval, 2014), these events occurred at the same time as a new era for social movements—the era of datafication (Meyer, 2024).

Datafication originally referred to “the collection, databasing, quantification and analysis of information, and the uses of these data as resources for knowledge production” (Flensburg and Lomborg, 2023; Mayer-Schönberger and Cukier, 2013). However, critical scholars subsequently contested this perception, contending that it promotes a view of data as a neutral resource that serves commercial interests and obscures underlying ideological assumptions (Boyd and Crawford, 2012; van Dijck, 2014).

While conventional movement logics have continued to function, datafication has had major consequences that have changed the stakes for social movements. These changes have challenged prominent theories on social movements, creating substantial gaps in the literature (Meyer, 2024). Recent reviews of literature in the field suggest that meaningful progress has been made in addressing these gaps over the last two decades (Caren et al., 2020; Meyer, 2024), but no systematic empirical research on the field’s evolution has yet been conducted.

Drawing on these observations, this study assesses the extent to which scholarship on social movements and social media (SMSM) has addressed the effects of datafication previously unforeseen by social movement scholars. The study also identifies underdeveloped areas where further research is needed and highlights promising methodological approaches with potential to investigate these areas. To do this, the study assesses three research questions:

*What research traditions have formed the canonical literature of SMSM scholarship over the last two decades of datafication?* (RQ1: Canonical literature). This question explores canonical traditions by focusing on the most cited studies in the field and investigates the extent to which scholars have engaged various traditions in meaningful dialogue.

*How have research themes in SMSM scholarship changed over the last two decades of datafication?* (RQ2: Thematic evolution). This question calls for an assessment of whether and how previously identified gaps in the literature related to datafication have been addressed as scholarship has evolved.

*How has SMSM scholarship evolved in its use of methodological approaches and methods over the last two decades of datafication*? (RQ3: Methods evolution). This question focuses on the shifts between traditional and emerging research methods.

We address these questions by applying literature mapping methods to analyze a dataset of 6701 academic studies published between 2005 and 2023. The metadata on these studies was extracted from the Web of Science using a search strategy that focused on research that investigates both social movements and social media. Our findings show that researchers in the field have thoroughly investigated most of the consequences of datafication, using several approaches and research methods, while drawing on multiple canonical research traditions.

In addition to confirming that most of the identified gaps have been addressed in the literature, our findings also identify under-researched areas related to a set of adverse consequences of social media on the organizational dynamics and long-term impacts of social movements. We further identify a shift in the methodological focus in the field from traditional research methods, such as qualitative interviews and surveys, to emerging techniques, including content-based computational analysis. Our analysis also highlights a consistent scarcity in experimental research that isolates causal mechanisms, and we point to experimental work in related fields that could advance this line of research. We conclude by discussing opportunities for next-step research that emerge from our findings, including the use of big data analysis as well as qualitative and experimental studies to address unresolved questions about social movements in the age of datafication.

## Delineating the field of SMSM

Social media emerged as a distinct concept in the 2010s, referring to the interactions facilitated by technological platforms with a particular set of communication features, such as creating a profile and connecting with other profiles (Burgess et al., 2018; Puschmann and Pentzold, 2021; Xenos et al., 2014). Since the emergence of social media, scholars have highlighted their complex role in society, including their emancipatory potential and their capacity to perpetuate power differences and oppressive social structures (Castells, 2009; Fuchs, 2024).

Definitions of social movements are diverse, ranging from a narrow focus on social movement organizations, which are formal organizations that operate to implement certain political preferences (McCarthy and Zald, 1977), to a broader definition including any action or effort people make aimed at changing an aspect of society, including individual activism, online expressions, and alternative lifestyles (Della Porta and Diani, 2015). The age of datafication has further complicated these definitions, diversifying the forms of participation in ways that make it harder to conceptualize and measure them (Ruess et al., 2023). In this study, we draw on a broad understanding of social movements that encompasses traditional social movement organizations (Amenta et al., 2010), individuals organizing or participating in social movement activities (van Stekelenburg and Klandermans, 2023), and hybrid and fluid practices of participation, including the use of hashtags and the sharing of personalized content (Jackson et al., 2020).

## Gaps identified in the field’s earlier stages

At the turn of the millennium, social movements scholarship was focused on the emergence of “the social movements society” (Meyer and Tarrow, 1997). This concept referred to the observation that social movement practices had become routine and prevalent across social sectors. In contrast to the stereotypical image of the fringe radical protestors in the 1960s, by the late 1990s, ordinary citizens from all walks of life and all opinions were using social movement tactics to advance their political agendas. This development coincided with the increased professionalization of social movements, which required more substantial resources to gain influence on public opinion through traditional media outlets.

Less than a decade later, the first major social media platforms were launched, ushering in the age of datafication. In his review of social movement studies in the age of datafication, Meyer (2024) observes that datafication fundamentally changed the stakes for social movements in ways that the scholarly literature has been slow to address. Meyer (2024) identifies the main changes datafication brought about, which the foundational theory of the social movement society had failed to foresee. We group the changes discussed in Meyer’s review into five broad developments:

1. Reducing the cost of participation;
2. Facilitating the flow of information;
3. Enabling top-down authoritarianism;
4. Aiding bottom-up authoritarianism; and
5. Imposing organizational constraints.

Developments (1) and (2) above have empowered movements by facilitating engagement and mobilization. The low cost of participation and the free flow of information have enabled ordinary citizens to easily take part in online movements while allowing movement organizers to bypass media gatekeepers and propagate messages beyond traditional geographic boundaries.

In contrast, developments (3), (4), and (5) demonstrate the less positive normative repercussions of datafication. Social media has contributed to top-down authoritarianism, as governments overcame their initial confusion regarding this new technology and developed effective ways to repress online dissent. Social media has also helped propagate bottom-up authoritarianism, strengthening far-right and anti-democratic messages and movements. By leaving individuals isolated in ideologically homogeneous communities, social media may have strengthened polarization and diminished the democratic dialogue that had previously mitigated shifts toward extremist discourses. Finally, datafication has imposed new challenges for social movements’ organizational dynamics and long-term impact. The lack of organizational institutionalization has led many movements to dissolve quickly and has weakened their internal accountability mechanisms, thereby compromising their effectiveness and durability.

Recent literature reviews of the field suggest that these unforeseen developments have largely been addressed over two decades of research (Caren et al., 2020; Meyer, 2024). Moving beyond dichotomous positive/negative views of social media (Foust and Hoyt, 2018; Kidd and McIntosh, 2016; Neumayer and Rossi, 2016), scholars have developed innovative research designs to study more complex questions, including what factors mediate between social media use and participation (Arslan et al., 2023; Boulianne et al., 2023; Fortunato and Pecoraro, 2022; Matthes et al., 2019), how repression and censorship are exercised on online networks (Chang, 2025; Earl et al., 2022), and how movements use social media for purposes beyond mobilization (Nhedzi and Azionya, 2025; Shim, 2024). But scholars have not yet explored how research on social media, as a whole, has evolved to address these repercussions of datafication.

Moreover, some scholars have argued that in its early stages, the field’s fascination with social media’s “newness” resulted in insufficient links to established research traditions and interdisciplinary dialogue (Kidd and McIntosh, 2016; Mattoni and Treré, 2014). These features also affected the field’s methodological scope, with some scholars arguing that social movements research has remained largely focused on traditional approaches and methods, such as interviews and surveys, whereas datafication offers opportunities to hone big data and innovative methods (Caren et al., 2020; Rohlinger, 2019). However, no systematic empirical study of the field’s canonical literature and methodological approaches has yet been conducted.

## Data and methods

We answer the three research questions using literature mapping and bibliometric analysis methods (van Eck and Waltman, 2010, 2014). Specifically, we used a lexical search to identify relevant research (Huang et al., 2015), and we created co-citation and co-occurrence maps based on the method of “visualization of similarities” formalized in the VOSviewer software (Traag et al., 2019; van Eck and Waltman, 2010), a tool commonly used to analyze bibliometric networks (Pan et al., 2018).

For the literature search, we sought to expand the scope of our database beyond that of existing systematic reviews and meta-analyses. These studies generally use generic terms like “social media,” “social networking sites,” and “collective action” (Hermann et al., 2023; Lin et al., 2024; Neumayer and Rossi, 2016; Pellegrini et al., 2020). Only a few studies include specific platforms in their search terms, limiting their searches to Facebook and Twitter (Amsalem and Zoizner, 2023; Boulianne, 2015; Lane et al., 2022). To expand coverage beyond these search approaches without compromising accuracy, we opted for a robust method developed in the context of STEM fields that uses objective thresholds to screen for additional frequent keywords and add specialized journals (Huang et al. 2015). This method includes three stages of search term development: a core lexical search, an expanded lexical search, and a specialized journals search (Huang et al. 2015). We sourced the data on scholarly works from the Web of Science (WoS) core collection. a bibliographic data source recognized for its high accuracy (Visser et al. 2021). While the Web of Science is a proprietary database with access restrictions that constrain data sharing, the quality of alternative open source databases is still being assessed, and further research is needed to analyze their accuracy and coverage (Alonso-Alvarez and van Eck, 2025; Culbert et al., 2025).

The core lexical search includes search terms based on existing literature reviews and scholarly definitions. For this initial stage, we selected keywords from prominent handbooks (Burgess et al., 2018; Della Porta and Diani, 2015). Our core search terms for social media included social media, Facebook, and Twitter, and for social movements included social movements, collective action, protest, and contentious politics. We used these terms to produce a core dataset.^1^

The expanded lexical search entailed broadening the search beyond what exists in the literature. It included retrieving frequent keywords from the core dataset and screening these using a hit ratio and a noise ratio. The hit ratio is a semi-automated estimate of the share of relevant studies retrieved by a candidate search term. The noise ratio is an estimate of the number of irrelevant results based on a manual check of a random selection of retrieved records. We extracted the 100 most frequent terms from the keywords and keywords plus fields of the core dataset (*N* = 7959). Removing spelling variations and generic terms, we composed a list of 81 candidate search terms and calculated their hit ratios. Following established thresholds (Huang et al. 2015), we included terms with a hit ratio of 70% or above. For candidate terms with a hit ratio between 30% and 70%, we calculated the noise ratio and included terms with a noise ratio below 50%. The expanded lexical search added 13 terms to our search term, including additional social media platforms such as Instagram and YouTube.^2^

The core and expanded lexical searches were applied to all journals indexed in the WoS core collection. To gain fuller coverage, we complemented these searches with a specialized journal search (Huang et al. 2015), which expands the lexical searches by adding all records in journals specializing in the fields of interest. We used common criteria in the field for selecting the journals (Liu et al. 2021; Shapira et al. 2017): (1) the journal’s purview is entirely within the scope of research on either social movements or social media; and (2) the journal is indexed in the WoS journal citations reports. Using these criteria, we identified three relevant journals: *Social Media + Society, Social Movement Studies*, and *Mobilization*.

To retrieve research at the intersection of the SMSM fields, we structured the final search term to combine one search term from each field. The resulting search term was:

((TS = (“social media” OR “Facebook” OR “Twitter” OR “sentiment analysis” OR “hashtag” OR “instagram” OR “youtube”) OR SO = “SOCIAL MEDIA SOCIETY”) AND (TS = (“social movement*” OR “social-movement*” OR “collective action” OR “protest*” OR “contentious politics” OR “Indignados” OR “occupy wall street” OR “occupy wall-street” OR “OWS” OR “activism” OR “Black Lives Matter” OR “Arab Spring” OR “mobilization” OR “mobilisation”) OR SO = (“SOCIAL MOVEMENT STUDIES” OR “MOBILIZATION”))) OR (TS = (“digital activism” OR “hashtag activism” OR “connective action”))

**Fig. 1:**
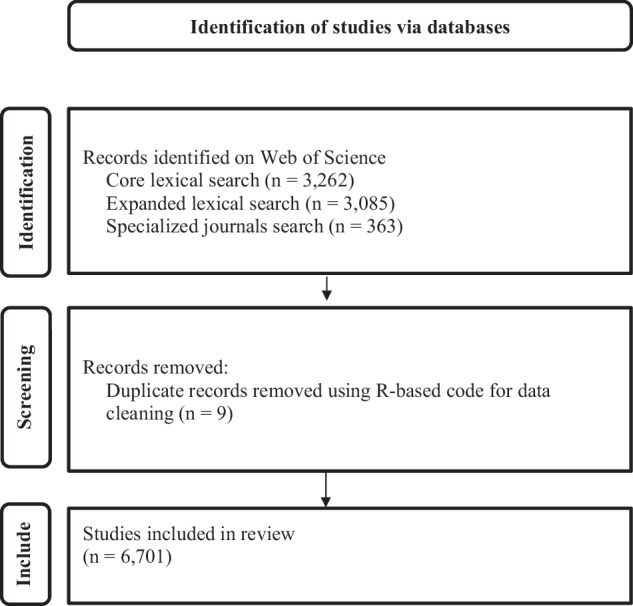
PRISMA chart.

This search produced a dataset of 6710 records.^3^ After removing duplicates, the dataset consisted of 6701 records. Figure 1 shows a PRISMA chart (Page et al., 2021) of our dataset construction process.

To answer RQ1 (Canonical literature), we analyzed co-citation relationships, limiting our analysis to around 100 of the most frequently cited references, which is common practice in the literature (Uthman et al., 2013). We used the most proximate threshold of 80 citations per reference, yielding 101 most cited references.^4^

To answer RQ2 (Thematic evolution), we analyzed co-occurrence relationships based on frequent keywords. To analyze the field’s temporal evolution, we followed standard practice in the literature to divide the dataset into distinct periods (Fils and van Eck, 2018; Liu et al., 2025), specifically: 2005–2009 (inception); 2010–2014 (emergence); 2015–2019 (consolidation); and 2020–2023 (maturation). Consistent with similar research in the field, we acknowledge that these descriptors are temporary heuristics, and we expect research to continue to mature and evolve in the coming years. The analysis for each period used the common threshold of terms appearing more than 15 times in the keyword fields (van Eck and Waltman, 2017).^5^

To answer RQ3 (Methods evolution), we conducted a temporal-historical bibliometric analysis (Walter and Ophir, 2024) to investigate the evolution of methods. Adapting this approach to SMSM scholarship, we compiled a list of keywords related to eleven categories of research methods.^6^ We then used analytical tools in R to perform automated detection of these keywords from the abstracts, keywords, and keywords plus fields in the dataset. We then analyzed the share of studies in each category by year.

Instead of relying on scholars’ subjective judgment, our analytical approach facilitates a systematic assessment of our research questions, using documented methods to allow for robust identification of changes in the scholarly landscape over time (Colavizza et al., 2021; Shoshan and Oser, 2025). These methods are commonly used in the natural sciences but have only recently been applied in social science research (Chipunza and Ntsalaze, 2025; Fontanella et al., 2024; Han et al., 2024).

The Supplemental Materials include additional information on data and methods. The replication files are available on Harvard Dataverse [10.7910/DVN/XL5L5R], including the replication protocols in VOSViewer (version 1.6.20), and analytical code in R (version 4.4.2).

## Results

### RQ1: Canonical literature

**Fig. 2:**
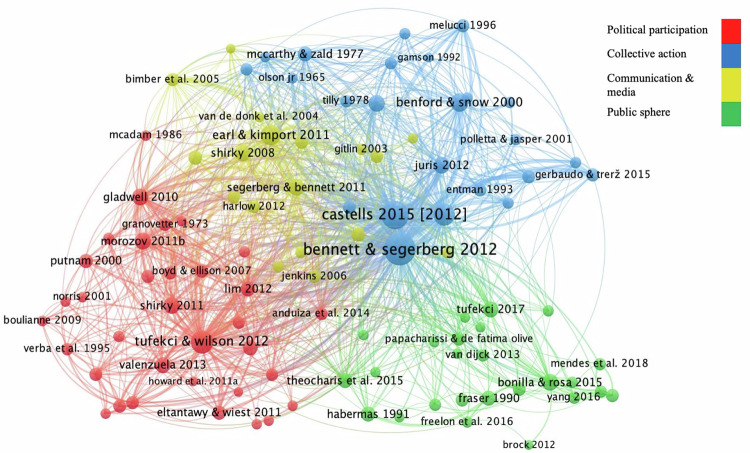
Canonical literature, co-citation analysis, *n* = 101 out of *N* = 229,241.

The co-citation relationships of the 101 most cited references in the dataset are represented in Fig. 2.

Based on a review of the titles and abstracts of the references in Fig. 2, we identified four research traditions that have informed research on SMSM over the last two decades:

1. Political Participation (red);
2. Collective Action (blue);
3. Communication and Media (yellow); and
4. Public Sphere (green).

The Political Participation canon (red) includes foundational theories focused on connecting individual-level political behavior, such as membership in voluntary organizations and voting, with macro-level democratic outcomes. It includes seminal works on social capital (Putnam, 2000), strong and weak ties (Granovetter, 1973; McAdam, 1986), and the connection between participation and representation (Verba et al., 1995). More recent studies in this canon have debated whether social media penetration enhances or hinders democratic processes (Howard and Hussain, 2013; Morozov, 2011) and have investigated how social media use affects political engagement (Boulianne, 2015; Tufekci and Wilson, 2012).

The Collective Action canon (blue) focuses on collective processes that enable individuals to act in concert. It includes seminal works on social movements’ emergence, mobilizing capabilities, and organizational dynamics, including resource mobilization (McCarthy and Zald, 1977), political opportunity structure (Tilly, 1978), framing processes (Snow et al., 1986), and collective identity (Polletta and Jasper, 2001). This cluster also includes more recent studies, such as Bennett and Segerberg’s (2012) seminal work on connective action, the most cited across the whole map, which defines the differences between traditional and digitally enabled action logics.

The Communication and Media canon (yellow) includes theories about the transition from top-down to networked communication systems and how this transition stimulates democratization processes (Cropf, 2008; Shirky, 2008). Another group of studies analyzed the uneasy relationship between social movements and traditional media outlets (Gamson and Wolfsfeld, 1993; Gitlin, 2003). Subsequent studies, focusing on hybrid media ecologies, have stressed the continuity of traditional media logics alongside the emergence of new ones (Chadwick, 2013; Harlow, 2012).

Finally, the Public Sphere canon (green) includes foundational political theories on the public sphere as a deliberative discursive space, one vital for democracy (Fraser, 1990; Habermas, 1991). Recent studies build upon these concepts to theorize networked forms of political action, such as hashtag activism, as a public space where dominant narratives are contested (Bonilla and Rosa, 2015), and new publics are constructed (Papacharissi, 2016; Tufekci, 2017).

This analysis shows that SMSM research over the last two decades has drawn upon four major research traditions associated with at least four disciplines: empirical political science (political participation and collective action), sociology (political participation and collective action), media studies (communication and media), and political theory (public sphere). All the clusters have numerous connections to one another, demonstrating that SMSM scholarship has brought foundational theories from all four disciplines into meaningful interdisciplinary dialogue.

### RQ2: Thematic evolution

**Fig. 3:**
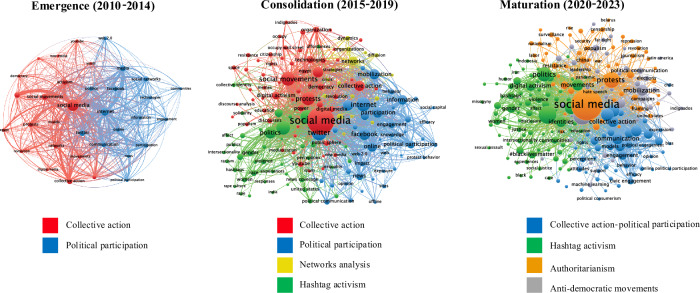
Main research themes in three periods, keywords co-occurrence analysis. Emergence (2010–2014): *n* = 34, *N* = 2011. Consolidation (2015–2019): *n* = 161, N = 6852. Maturation (2020–2023): *n* = 246, *N* = 9802.

Figure 3 presents our analysis of co-occurrence relationships between keywords in each period for which sufficient data are available. The “Inception” period (2005–2009) is not included in this analysis because of its small sample size (*n* = 11), which does not allow for a meaningful analysis of themes.

Our co-occurrence analysis of data in the emergence period (2010–2014) shows that studies in these years focused on the normatively positive consequences of datafication, which arose from the reduction of participation costs and the removal of barriers to spreading information. The two clusters in the map represent two approaches for studying these developments. The first cluster, labeled political participation (blue), focused on engagement based on individual-level parameters and studied whether and how access to and use of social media enhanced the probability of becoming politically engaged (Kruikemeier et al., 2014; Oser et al., 2013). In contrast, the second cluster, labeled collective action (red), focused on the organizational level, exploring how movements use social media to spread their message and to mobilize (Micó and Casero-Ripollés, 2014; Segerberg and Bennett, 2011).

In the consolidation period (2015–2019), the two earlier clusters of political participation (blue) and collective action (red) expanded, representing both quantitative growth and more nuanced and complex research. In research on political participation, meta-analyses found variations in the relationships between social media use and political engagement depending on the context, platforms, and forms of engagement (Boulianne, 2018; Skoric et al., 2016). Meanwhile, studies on collective action made headway in elaborating on the concept of connective action (Bennett and Segerberg, 2012), highlighting the distinctive organizational features of networked logics compared with traditional ones (Theocharis, 2015; Vicari and Cappai, 2016).

Another prominent theme in the consolidation period is the free flow of information. Interest in this theme expanded to include two distinct clusters, representing two approaches for studying this phenomenon: network analysis (yellow) and hashtag activism (green). Network analysis studies focused on using social media data to trace network structure and dissemination channels. Hashtag activism research studied digital movements by theorizing hashtags as discursive tools constituting a public space (Bonilla and Rosa, 2015), where groups construct counternarratives and counter-publics (Bosch, 2017; Papacharissi, 2016). Overall, the consolidation period established theoretically and methodologically complex research on SMSM, while the thematic focus remained on how social media empowers movements and facilitates engagement and mobilization.

In the maturation period (2020–2023), scholars began to meaningfully address the normatively negative ramifications of datafication, including top-down and bottom-up authoritarianism (Meyer, 2024). As the thematic map of the maturation period shows, these two themes are represented by two new clusters, labeled authoritarianism (orange) and anti-democratic movements (gray). The authoritarianism cluster includes research on how governments developed effective ways to surveil, censor, and repress online dissent (Earl et al., 2022; Gohdes, 2020; Golovchenko, 2020), as well as the spread of disinformation and misinformation (Teng et al., 2022; Valenzuela et al., 2021). The cluster focused on anti-democratic movements (gray) involves research on how social media helps to spread xenophobic and nationalist frames (Alonso-Muñoz and Casero-Ripollés, 2023; Flew and Iosifidis, 2020) and the related theme of left-right polarization (Freelon et al., 2020; Knuepfer et al., 2022).

Another feature of the maturation period (2020–2023) is that the field’s more established streams of research—political participation and collective action—merged into one cluster (blue). This development represents enhanced synergies in these fields and a shared interest in robust methods to identify increasingly complex and often reciprocal relationships between social media use, engagement, and mobilization (Ansar and Khaled, 2023; Bonilla and Tillery, 2020; Oser et al., 2022). Research on hashtag activism (green cluster) also expanded to include sophisticated analyses of visual data, highlighting both the visual practices of resistance and how repressive representations permeate visual content online (Eriksson Krutrök and Åkerlund, 2023; Klein et al., 2022; McKenna and Chughtai, 2020).

Taken together, the results of the co-occurrence analysis show that over the past two decades, research has meaningfully investigated most themes identified in Meyer’s (2024) review. However, one main theme—social media’s adverse effects on movement organization and long-term impact—has received limited attention.

### RQ3: Methods evolution

**Fig. 4:**
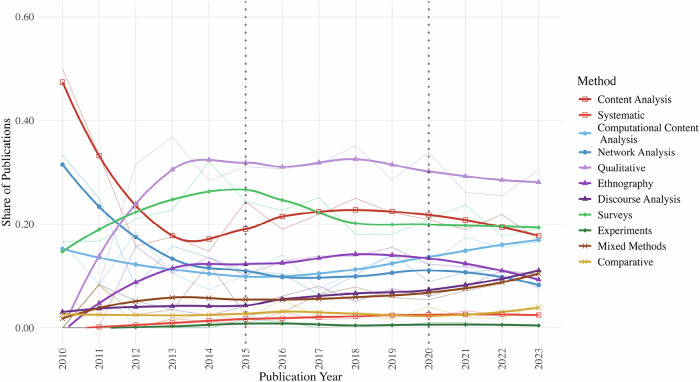
Methods evolution. Share of methods in abstracts over time, out of all publications mentioning at least one method, *n* = 2737 out of *N* = 6701.

The methodological trends in the three periods for which sufficient data exist are represented in Fig. 4.

At the beginning of the emergence period (2010–2014), prevalent methodologies included content analysis and network analysis. We suggest that this methodological focus is a result of the massive amount of available data generated by the mass protest cycles of 2010–2012. This data lent itself easily to the well-developed techniques of computational network analysis and non-computational content analysis, which researchers can conduct relatively quickly compared to interviews or ethnography.

However, by the end of the emergence period, the share of network and content analysis studies had declined in favor of more traditional methods, including surveys and qualitative methods. We also see the increased use of systematic reviews, meta-analyses, and experiments. Several experiment-based studies during this period offer innovations in evaluating causal relationships between social media activity and political participation, representing a crucial methodological advance (Bond et al., 2012; Lim and Golan, 2011).

In the consolidation period (2015–2019), methodological trends remained relatively stable, with traditional methods, such as surveys and qualitative methods, dominating the field. The maturation period (2020–2023) is marked by a different trend: a moderate decline in the share of these traditional methods combined with a noticeable rise in the share of computational content analysis methods, which include techniques such as topic modeling, sentiment analysis, and natural language processing. There is also an upward trend in the mixed methods category, which includes techniques such as triangulation and process tracing.

## Discussion

We summarize our results in Table 1.

**Table 1:** Summary of findings.

RQ1: Canonical literature				
**Canon**	Political participation	Collective action	Communication and media	Public sphere
**Discipline**	Empirical political science, sociology	Empirical political science, sociology	Communication and media	Political theory

RQ2: Thematic evolution				
Development	Main themes	Research approaches (clusters)		
		Emergence 2010–2014	Consolidation 2015–2019	Maturation 2020–2023
**Low cost of participation**	Mobilization	Collective action	Collective action	Collective action-political participation
	Engagement	Political participation	Political participation	
**Dissemination of information**	Diffusion networks	Collective action	Networks analysis	Collective action-Political participation
	Frames publics narratives	NA	Hashtag activism	Hashtag activism
**Top-down authoritarianism**	Repression censorship surveillance disinformation	NA	NA	Authoritarianism
**Bottom-up authoritarianism**	Far right nationalism xenophobia polarization	NA	NA	Anti-democratic movements
**Organizational constraints**	Dissolution Weak accountability impact	NA	NA	NA

RQ3: Methods evolution				
	Emergence 2010–2014	Consolidation 2015–2019		Maturation 2020–2023
**Upward trends**	Qualitative methods and surveys experiments and systematic reviews	Stable		Computational content analysis mixed methods
**Downward trends**	Networks and content analysis	Stable		Qualitative methods, surveys, content analysis

The mapping of two decades of scholarship on the intersection of social media and social movements has clarified that most of the unforeseen developments of datafication (Meyer, 2024) have been thoroughly researched using multiple theoretical and methodological approaches. The investigation of our first research question (RQ1: Canonical literature) shows that despite earlier claims about the disconnect of SMSM scholarship from established canons, the field has drawn on four areas of canonical literature: political participation, collective action, communication and media studies, and the public sphere. In contrast to prior concerns about the lack of interdisciplinary dialogue in SMSM research, our co-citation analysis shows that scholars have substantially integrated these traditions.

Our analysis based on the second research question (RQ2: Thematic evolution) shows that four of the five broad developments brought about by datafication (Meyer, 2024) have been thoroughly studied, including the low cost of participation, the rapid dissemination of information, and the rise of top-down and bottom-up authoritarian processes. Scholars have investigated these developments through several lenses: individual-level parameters of political participation; organizational-level analyses of collective action and mobilization; network analysis of the dissemination of information and movement frames; content analysis of hashtag activism as a new public sphere; and the rise of top-down and bottom-up anti-democratic processes.

However, our findings show a paucity of research into developments related to social media’s adverse effects on movements’ organizational dynamics, including their durability and internal checks and balances, as well as the effect of social media on movements’ long-term impact. We identified several studies that focus on the negative effects of social media on movements’ organizational dynamics, including the debilitating effect of profit-driven algorithms and the lack of institutionalized infrastructure (Etter and Albu, 2021; Shahin and Ng, 2022). However, the absence of thematic clusters that focus on these themes in the co-occurrence maps suggests that these themes have been under-researched compared with the other themes we identified. This finding may be a result of scholars’ tendency to focus on high-profile case studies at the expense of studying social movement initiatives that have been hampered or sidelined. More research is needed to understand how social media may negatively affect the organizational dynamics and long-term political impact of contemporary movements.

Our analysis of the evolution of methods in SMSM research, regarding our third research question (RQ3: Methods evolution), shows a shift from an initial focus on traditional methods, such as focus groups, interviews, and surveys, toward a rising interest in computational content analysis methods, such as topic modeling and sentiment analysis. These developments offer important opportunities for SMSM scholars to delve deeper into the informational content and affective tone of messages on social media.

Notably, the main persistent gaps in the literature that we identified—the study of constraints on movements’ organizational dynamics and long-term impact in the age of datafication—require multi-method approaches to break new ground. A combination of methodological tools is needed to study the dissolution of movements or the dynamics of internal dissent, including in-depth qualitative analyses, the quantitative analysis of event data, and other traditional tools.

Additionally, the study of movements’ long-term impact requires methods that facilitate causal analysis. While big data has enabled scholars to implement innovative research designs to study causal relationships in political behavior (Bruns et al., 2013; Guess et al., 2023; Mercea et al., 2024) and public discourse (Dunivin et al., 2022; Freelon et al., 2018; Levy and Mattsson, 2021), our findings support prior observations that studies of movements’ political outcomes remain scarce (Caren et al., 2020). Some scholars have argued that SMSM research could benefit from an increased focus on experimental research to address these gaps (Rohlinger, 2019). Our analysis shows that this suggestion remains relevant: the share of experimental studies in SMSM research has not grown meaningfully over the years, remaining below two percent throughout the entire period under investigation. This finding indicates that SMSM scholarship could benefit from a greater focus on experimental research designs to examine topics such as movements’ political impact in the age of datafication. Recent methodological innovations in experiments on social media provide practical guides for advancing this line of research (Aridor et al., 2025).

## Conclusion

The evolution of social movements in the age of datafication has challenged prevailing social movement theories (Meyer, 2024). Datafication led to five broad developments that contradicted earlier expectations. While it empowered movements by (1) reducing the cost of participation and (2) removing barriers to disseminating information, it also had consequences that are less normatively positive, including the rise of (3) top-down authoritarianism and (4) bottom-up authoritarianism, as well as (5) new constraints on movements’ organizational dynamics and long-term impact.

The mapping of scholarship on the intersection of social media and social movements through two decades of datafication has shown that the first four developments have been thoroughly researched with a focus on five aspects: individual-level parameters of participation; organizational-level parameters of mobilization; the structure of networks that propagate movement frames, the new public sphere constituted by hashtag activism; and the flourishing of authoritarian practices and anti-democratic movements. In addition, our analysis shows that contrary to earlier critiques of the field, scholars have conducted research on these developments by meaningfully integrating four established canonical traditions: political participation, collective action, communication, and media studies. and the public sphere.

However, this study also highlights the scarcity of research on questions related to the varied consequences of social media for movements’ organizational dynamics and long-term impact. The lack of institutionalization may lead to the quick dissolution of movements, while easy desertion could lead to a decline in accountability mechanisms (Meyer, 2024). Both processes could undermine the potential for social movements to generate and sustain long-term political outcomes. Our findings indicate that these themes would benefit from additional research.

Methodologically, our findings show important contributions stemming from the increased use of computational methods, which have enabled scholars to investigate the affective impact of social media frames on an unprecedented scale (Calvo et al., 2025; Eady et al., 2023; Vu et al., 2021; Wu et al., 2024). Our findings also contribute to concerns in the broader literature about the risks and drawbacks associated with increased reliance on big data analysis (Boyd and Crawford, 2012; Ozkula et al., 2023). These include the risk of treating minority and lower-status groups as outliers (Hargittai, 2020; Kreiss and McGregor, 2024) and the concerning decline in data access, with platforms closing APIs and charging access fees (Davidson et al., 2023; de Vreese and Tromble, 2023; Freelon, 2018).

Additional methods and tools are required to address the questions about SMSM that this study identified as needing more research. Movements’ internal dynamics may be adequately analyzed using in-depth qualitative research, while identifying the causal links between movements’ online activity and long-term political outcomes may require more experimental research. Overall, our findings point to the importance of scholars’ continued use of both traditional and emerging methodological tools to advance ground-breaking research on the changing environment of social movements, including social media’s varied effects, as the age of datafication continues to evolve.

## Supplementary information

Supplementary Material

## Data Availability

The data that support the findings of this study were extracted from the Web of Science, which is a subscription-based service provided by Clarivate. Access to the Web of Science is restricted and requires an institutional or individual subscription. We have received permission from the Web of Science to publicly share data used in our analysis for all metadata fields except for the “Abstracts” field, which is subject to licensing restrictions from publishers. Our replication files, posted to the Harvard Dataverse [10.7910/DVN/XL5L5R] (Shoshan et al. 2026), provide guidance for replicating our findings in accordance with Clarivate’s data usage permission.

## Footnotes

1 Downloaded on October 29, 2023.

2 Non-Western platforms like TikTok and Telegram did not pass the objective threshold of occurrences in the core dataset. We performed a new search on the Web of Science on November 27, 2024, on social movements, finding that until 2023, only 49 social movement studies related to TikTok and 69 studies related to Telegram. These platforms will potentially reach objective empirical thresholds in the future as research expands.

3 Downloaded on January 1, 2024.

4 For the list of references by cluster, see Section D of the Supplemental Materials.

5 For the list of common keywords by period, see Section E of the Supplemental Materials.

6 For the list of methodological keywords by category, see Section F of the Supplemental Materials.
