# Supplementary material for: Social movements and social media: the evolution of scholarship in the age of datafication
Source: Humanit Soc Sci Commun. 2026 Apr 28;13(1):1024. doi: 10.1057/s41599-026-07128-9 (PMC13333502; doi:10.1057/s41599-026-07128-9)
Supplement: Supplementary file 1 — Supplementary Material [file 41599_2026_7128_MOESM1_ESM.docx]

Supplemental Materials

Section A: Search Method

Section B: Dataset Timeline

Section C: Analytic Settings for Maps

Section D: Co-citation References by Cluster

Section E: Co-occurrence Keywords Data

Section F: Computational Historical Analysis of Methods

References for Supplemental Materials

**Section A: Search Method**

The search method used in this study is based on the model developed by Huang et al. (2015) to delineate research on an emerging technology. The search term was built in three stages: core lexical search, expanded lexical search, and specialized journals search.

1. Core search

Table A1: Core lexical search

| **Expert sources** | **Core search term** | **Results** | **Date performed** |
| --- | --- | --- | --- |
| Burgess et al., 2018  Della Porta & Diani, 2015 | TS=(“social media” OR “Facebook” OR “Twitter”) AND TS=(“social movement*” OR “social-movement*” OR “collective action” OR “protest*” OR “contentious politics”) | 3,262 | October 29, 2023 |

1. Expanded lexical search

For the expanded search, we compiled a list of 81 candidate terms based on the 100 most frequent keywords and keywords plus in the core dataset (N=7,959), removing terms already included in the core search, generic words, spelling variations, and single/plural variations. For each candidate term, we determined whether it was related to the social media field (category A), social movements field (category B), or combined (category C).

We then calculated the hit ratio (Huang et al., 2015) for each candidate term, adjusting the formula to scholarship at the intersection of two fields. The adapted formula calculates the share of the results retrieved by a candidate term from one category in conjunction with at least one term from the other category that are already included in the core search (“hits”), out of the total number of results that the combined search yielded:

Hit ratio = X&Y/Y

Where

X=results of core search

Y=results obtained from the combination of a candidate term from one category and one or more of the core search terms of the second category

For candidate terms in category C (related to both social media and social movements), there was no need to include core search terms from the other category. Hence, their calculation was as follows:

Hit ratio = X&Y/Y

Where

X=results of core search

Y=results obtained by the candidate search term

Thresholds used (Huang et al., 2015):

Hit ratio >=70%: Accept

Hit ratio <=30%: Reject

30%>Hit ratio>70%: Manual check (noise ratio)

Noise ratio=<50%: Accept

For the manual check of candidate terms in the middle range, we created a random sample of 10% of records that constituted potential noise, that is, they were retrieved by the combined search of the candidate term and one of the core terms of the other category, but were not included in the results of the core search (Y not X). Based on canonical definitions of social media and social movements from the literature, we trained a coder to determine whether each record in the sample is relevant for the field.

Table A2: Expanded lexical search

| **Candidate term** | **Category** | **Hit ratio** | **Noise ratio** | **Decision** |
| --- | --- | --- | --- | --- |
| political protest | B | 1.00 | NA | All results included in core search |
| protest behavior OR protest behaviour | B | 1.00 | NA | All results included in core search |
| indignados | B | 0.88 | NA | Include |
| sentiment analysis | A | 0.86 | NA | Include |
| occupy wall-street OR occupy wall street or OWS | B | 0.86 | NA | Include |
| hashtag | A | 0.84 | NA | Include |
| instagram | A | 0.81 | NA | Include |
| youtube | A | 0.62 | 0.13 | Include |
| black lives matter | B | 0.58 | 0.20 | Include |
| internet use | A | 0.58 | 0.70 | Exclude |
| digital media | A | 0.55 | 0.38 | Include |
| collective identity | B | 0.55 | 0.60 | Exclude |
| big data | A | 0.53 | 0.88 | Exclude |
| machine learning | A | 0.51 | 1.00 | Exclude |
| connective action | C | 0.51 | 0.13 | Include |
| online | A | 0.46 | 0.76 | Exclude |
| arab spring | B | 0.46 | 0.38 | Include |
| mobilization or mobilisation | B | 0.45 | 0.46 | Include |
| media | A | 0.43 | 0.88 | Exclude |
| activism | B | 0.41 | 0.14 | Include |
| internet | A | 0.41 | 0.63 | Exclude |
| movement* | B | 0.39 | 0.50 | Include |
| social network analysis | A | 0.35 | 0.88 | Exclude |
| network analysis | A | 0.33 | 1.00 | Exclude |
| hashtag activism | C | 0.33 | 0.00 | Include |
| digital activism | C | 0.31 | 0.00 | Include |
| content analysis | A | 0.29 | NA | Exclude |
| social network* | A | 0.29 | NA | Exclude |
| online activism | C | 0.26 | NA | Exclude |
| communication* | A | 0.26 | NA | Exclude |
| egypt | B | 0.24 | NA | Exclude |
| political participation OR political-participation | B | 0.21 | NA | Exclude |
| hong kong | B | 0.21 | NA | Exclude |
| information | A | 0.19 | NA | Exclude |
| revolution | B | 0.18 | NA | Exclude |
| resistance | B | 0.17 | NA | Exclude |
| network* | A | 0.17 | NA | Exclude |
| technology | A | 0.17 | NA | Exclude |
| feminism | B | 0.17 | NA | Exclude |
| framing | B | 0.15 | NA | Exclude |
| democracy | B | 0.15 | NA | Exclude |
| civic engagement | B | 0.14 | NA | Exclude |
| public sphere | B | 0.14 | NA | Exclude |
| politics | B | 0.14 | NA | Exclude |
| censorship | B | 0.14 | NA | Exclude |
| violence | B | 0.12 | NA | Exclude |
| citizenship | B | 0.11 | NA | Exclude |
| populism | B | 0.09 | NA | Exclude |
| participation | B | 0.08 | NA | Exclude |
| conflict | B | 0.08 | NA | Exclude |
| discourse | B | 0.08 | NA | Exclude |
| race | B | 0.08 | NA | Exclude |
| identit* | B | 0.07 | NA | Exclude |
| political communication | B | 0.07 | NA | Exclude |
| Power | B | 0.07 | NA | Exclude |
| affordance* | B | 0.06 | NA | Exclude |
| climate change | B | 0.06 | NA | Exclude |
| coverage | B | 0.06 | NA | Exclude |
| leadership | B | 0.06 | NA | Exclude |
| expression | B | 0.06 | NA | Exclude |
| diffusion | B | 0.05 | NA | Exclude |
| youth | B | 0.05 | NA | Exclude |
| organization* | B | 0.05 | NA | Exclude |
| journalism | B | 0.05 | NA | Exclude |
| culture | B | 0.04 | NA | Exclude |
| news | B | 0.04 | NA | Exclude |
| surveillance | B | 0.04 | NA | Exclude |
| women | B | 0.04 | NA | Exclude |
| engagement | B | 0.03 | NA | Exclude |
| china | B | 0.03 | NA | Exclude |
| efficacy | B | 0.03 | NA | Exclude |
| opinion | B | 0.03 | NA | Exclude |
| gender | B | 0.02 | NA | Exclude |
| emotion* | B | 0.02 | NA | Exclude |
| community | B | 0.02 | NA | Exclude |
| exposure | B | 0.02 | NA | Exclude |
| behavior OR behaviour | B | 0.02 | NA | Exclude |
| attitude* | B | 0.02 | NA | Exclude |
| trust | B | 0.02 | NA | Exclude |
| perception* | B | 0.01 | NA | Exclude |
| covid-19 | B | 0.01 | NA | Exclude |

1. Specialized journals

Table A3: Specialized journals search.

| **Criteria for selection** | **Journals on social media** | **Journals on social movements** |
| --- | --- | --- |
| 1. The journal’s purview is entirely within the scope of research on either social movements or social media 2. The journal is indexed in the WoS journal citations reports. | *Social Media + Society* | *Social Movements Studies*  *Mobilization* |

1. Final search

Table A4: Details on final search.

| **Boolean search term** | **Results** | **Duplicates** | **Dataset size** | **Date performed** |
| --- | --- | --- | --- | --- |
| ((TS=(“social media” OR “Facebook” OR “Twitter” OR “sentiment analysis” OR “hashtag” OR “instagram” OR “youtube”) OR SO=“SOCIAL MEDIA SOCIETY”) AND (TS=(“social movement*” OR “social-movement*” OR “collective action” OR “protest*” OR “contentious politics” OR “Indignados” OR “occupy wall street” OR “occupy wall-street” OR “OWS” OR “activism” OR “Black Lives Matter” OR “Arab Spring” OR “mobilization” OR “mobilisation”) OR SO=(“SOCIAL MOVEMENT STUDIES” OR “MOBILIZATION”))) OR (TS=(“digital activism” OR “hashtag activism” OR “connective action”)) | 6,710 | 10 | 6,701 | January 1, 2024 |

**Section B: Dataset Timeline**

Figure B1: Average annual publication rate by period.


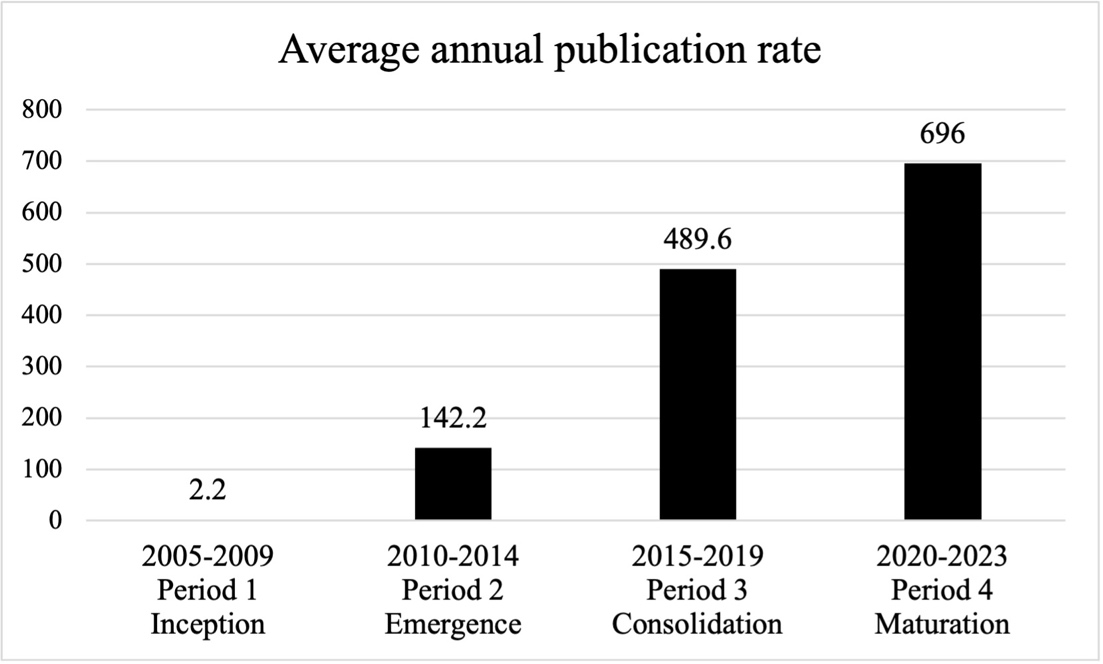


**Section C: Analytic Settings for Maps**

Following recommended procedure (van Eck & Waltman, 2023), we used the following map settings on VOSViewer:

1. Thesaurus files.
2. Full counting option.
3. After testing several cluster resolution parameters, using the 0.9 resolution parameter, which gave the most coherent and useful clustering.
4. Minimum number of 3 keywords per cluster.
5. Adjusting cluster colors and map orientation to facilitate chronological comparison of periods.

Additional resources on VOSviewer mapping and clustering techniques:

1. van Eck, N. J., Waltman, L., Dekker, R., & Van Den Berg, J. (2010). A comparison of two techniques for bibliometric mapping: Multidimensional scaling and VOS. *Journal of the American Society for Information Science and Technology*, *61*(12), 2405–2416. <https://doi.org/10.1002/asi.21421>
2. Waltman, L., van Eck, N. J., & Noyons, E. C. M. (2010). A unified approach to mapping and clustering of bibliometric networks. *Journal of Informetrics*, *4*(4), 629–635. <https://doi.org/10.1016/j.joi.2010.07.002>

Additional resources for creating literature maps on VOSviewer:

1. van Eck, N. J., & Waltman, L. 2023. *Manual for VOSviewer version 1.6.20*. Univeristeit Leiden. <https://www.vosviewer.com/documentation/Manual_VOSviewer_1.6.20.pdf>.
2. Shoshan, A., & Oser, J. (2025). Visualizing Scientific Landscapes: A Powerful Method for Mapping Research Fields. *PS: Political Science & Politics*, *58*(1), 147–154. <https://doi.org/10.1017/S1049096524001057>

**Section D: Co-citation References by Cluster**

Table D1 shows the list of the 101 most cited references in the dataset, the number of citations of each reference, and the thematic cluster to which it belongs.

Table D1: Most cited references by cluster.

| **Cited reference** | **Citations** | **Cluster** | **Full reference** |
| --- | --- | --- | --- |
| tufekci & wilson 2012 | 390 | Political participation | Tufekci, Z., & Wilson, C. (2012). Social media and the decision to participate in political protest: Observations from Tahrir Square. *Journal of Communication*, *62*(2), 363–379.‏ |
| morozov 2011b | 220 | Political participation | Morozov, E. (2011). The Net Delusion: The Dark Side of Internet Freedom. Public Affairs. |
| gladwell 2010 | 217 | Political participation | Gladwell, M. (2010, October 4). Small change: Why the revolution won’t be tweeted. The New Yorker, 4. ‏ |
| shirky 2011 | 208 | Political participation | Shirky, C. (2011). The political power of social media: Technology, the public sphere, and political change. *Foreign Affairs*, *90*(1), 28–41. |
| valenzuela 2013 | 187 | Political participation | Valenzuela, S. (2013). Unpacking the use of social media for protest behavior: The roles of information, opinion expression, and activism. *American Behavioral Scientist*, *57*(7), 920–942.‏ |
| lim 2012 | 183 | Political participation | Lim, M. (2012). Clicks, cabs, and coffee houses: Social media and oppositional movements in Egypt, 2004–2011. *Journal of Communication*, *62*(2), 231–248.‏ |
| lotan et al. 2011 | 164 | Political participation | Lotan, G., Graeff, E., Ananny, M., Gaffney, D., & Pearce, I. (2011). The Arab Spring. The revolutions were tweeted: Information flows during the 2011 Tunisian and Egyptian revolutions. *International Journal of Communication*, *5*, 31.‏ |
| eltantawy & wiest 2011 | 152 | Political participation | Eltantawy, N., & Wiest, J. B. (2011). The Arab Spring. Social media in the Egyptian revolution: reconsidering resource mobilization theory. *International Journal of Communication*, *5*, 18.‏ |
| putnam 2000 | 148 | Political participation | Putnam, R. D. (2000). *Bowling alone: The collapse and revival of American community*. Simon and Schuster.‏ |
| boyd & ellison 2007 | 141 | Political participation | Boyd, D. M., & Ellison, N. B. (2007). Social network sites: Definition, history, and scholarship. *Journal of Computer‐mediated Communication*, *13*(1), 210–230.‏ |
| de zúñiga hg, 2012 | 139 | Political participation | Gil de Zúñiga, H., Jung, N., & Valenzuela, S. (2012). Social media use for news and individuals' social capital, civic engagement and political participation. *Journal of Computer-mediated Communication*, *17*(3), 319–336.‏ |
| king et al. 2013 | 139 | Political participation | King, G., Pan, J., & Roberts, M. E. (2013). How censorship in China allows government criticism but silences collective expression. *American Political Science Review*, *107*(2), 326–343.‏ |
| boulianne 2015 | 131 | Political participation | Boulianne, S. (2015). Social media use and participation: A meta-analysis of current research. *Information, Communication & Society*, *18*(5), 524–538.‏ |
| granovetter 1973 | 131 | Political participation | Granovetter, M. S. (1973). The strength of weak ties. *American Journal of Sociology*, *78*(6), 1360–1380.‏ |
| kaplan & haenlein 2010 | 128 | Political participation | Kaplan, A. M., & Haenlein, M. (2010). Users of the world, unite! The challenges and opportunities of Social Media. *Business Horizons*, *53*(1), 59–68.‏ |
| valenzuela et al. 2012b | 128 | Political participation | Valenzuela, S., Arriagada, A., & Scherman, A. (2012). The social media basis of youth protest behavior: The case of Chile. *Journal of Communication*, *62*(2), 299–314.‏ |
| howard & hussain 2013 | 125 | Political participation | Howard, P. N., & Hussain, M. M. (2013). *Democracy's fourth wave?: digital media and the Arab Spring*. Oxford University Press.‏ |
| anduiza et al. 2014 | 119 | Political participation | Anduiza, E., Cristancho, C., & Sabucedo, J. M. (2014). Mobilization through online social networks: the political protest of the indignados in Spain. *Information, Communication & Society*, *17*(6), 750–764.‏ |
| verba et al. 1995 | 116 | Political participation | Verba, S., Schlozman, K. L., & Brady, H. E. (1995). *Voice and equality: Civic voluntarism in American politics*. Harvard University Press.‏ |
| gonzález-bailón s, 2011 | 112 | Political participation | González-Bailón, S., Borge-Holthoefer, J., Rivero, A., & Moreno, Y. (2011). The dynamics of protest recruitment through an online network. *Scientific Reports*, *1*(1), 1–7.‏ |
| bond et al. 2012 | 106 | Political participation | Bond, R. M., Fariss, C. J., Jones, J. J., Kramer, A. D., Marlow, C., Settle, J. E., & Fowler, J. H. (2012). A 61-million-person experiment in social influence and political mobilization. *Nature*, *489*(7415), 295–298.‏ |
| van zomeren et al. 2008 | 105 | Political participation | Van Zomeren, M., Postmes, T., & Spears, R. (2008). Toward an integrative social identity model of collective action: a quantitative research synthesis of three socio-psychological perspectives. *Psychological Bulletin*, *134*(4), 504.‏ |
| howard & hussain 2011 | 102 | Political participation | Howard, P. N., & Hussain, M. M. (2011). The role of digital media. *Journal of Democracy*, *22*, 35.‏ |
| wolfsfeld et al. 2013 | 92 | Political participation | Wolfsfeld, G., Segev, E., & Sheafer, T. (2013). Social media and the Arab Spring: Politics comes first. *The International Journal of Press/Politics*, *18*(2), 115–137.‏ |
| mcadam 1986 | 89 | Political participation | McAdam, D. (1986). Recruitment to high-risk activism: The case of freedom summer. *American Journal of Sociology*, 92(1), 64–90.‏ |
| norris 2001 | 89 | Political participation | Norris, P. (2001). *Digital divide: Civic engagement, information poverty, and the Internet worldwide*. Cambridge University Press.‏ |
| boulianne 2009 | 86 | Political participation | Boulianne, S. (2009). Does Internet use affect engagement? A meta-analysis of research. *Political Communication*, *26*(2), 193–211.‏ |
| khondker 2011 | 85 | Political participation | Khondker, H. H. (2011). Role of the new media in the Arab Spring. *Globalizations*, *8*(5), 675–679.‏ |
| christensen 2011b | 83 | Political participation | Christensen, H. S. (2011). Political activities on the Internet: Slacktivism or political participation by other means?. *First Monday*, *16*(2). |
| de zúñiga hg, 2014 | 83 | Political participation | Gil de Zúñiga, H., Molyneux, L., & Zheng, P. (2014). Social media, political expression, and political participation: Panel analysis of lagged and concurrent relationships. *Journal of Communication*, 64(4), 612–634.‏ |
| howard 2010 | 82 | Political participation | Howard, P. N. (2010). *The digital origins of dictatorship and democracy: Information technology and political Islam*. Oxford University Press.‏ |
| yang 2009 | 82 | Political participation | Yang, G. (2009). *The power of the Internet in China: Citizen activism online*. Columbia University Press.‏ |
| howard et al. 2011a | 81 | Political participation | Howard, P. N., Duffy, A., Freelon, D., Hussain, M. M., Mari, W., & Maziad, M. (2011). Opening closed regimes: What was the role of social media during the Arab Spring? *SSRN*. https://doi.org/10.2139/ssrn.2595096‏ |
| jost et al. 2018 | 81 | Political participation | Jost, J. T., Barberá, P., Bonneau, R., Langer, M., Metzger, M., Nagler, J., ... & Tucker, J. A. (2018). How social media facilitates political protest: Information, motivation, and social networks. *Political Psychology, 39*(S1), 85–118. |
| bonilla & rosa 2015 | 226 | Public sphere | Bonilla, Y., & Rosa, J. (2015). #Ferguson: Digital protest, hashtag ethnography, and the racial politics of social media in the United States. *American Ethnologist*, *42*(1), 4–17. |
| tufekci 2017 | 196 | Public sphere | Tufekci, Z. (2017). *Twitter and tear gas: The power and fragility of networked protest*. Yale University Press. |
| fraser 1990 | 173 | Public sphere | Fraser, N. (1990). Rethinking the public sphere: A contribution to the critique of actually existing democracy. *Social Text*, *25/26*, 56–80. |
| theocharis et al. 2015 | 169 | Public sphere | Theocharis, Y., Lowe, W., Van Deth, J. W., & García-Albacete, G. (2015). Using Twitter to mobilize protest action: Online mobilization patterns and action repertoires in the Occupy Wall Street, Indignados, and Aganaktismenoi movements. *Information, Communication & Society, 18*(2), 202–220. ‏ |
| habermas 1991 | 166 | Public sphere | Habermas, J. (1991). *The structural transformation of the public sphere: An inquiry into a category of bourgeois society*. MIT Press.‏ |
| braun & clarke 2006 | 141 | Public sphere | Braun, V., & Clarke, V. (2006). Using thematic analysis in psychology. *Qualitative Research in Psychology, 3*(2), 77–101.‏ |
| papacharissi 2016 | 138 | Public sphere | Papacharissi, Z. (2016). Affective publics and structures of storytelling: Sentiment, events and mediality. *Information, Communication & Society*, *19*(3), 307–324. |
| papacharissi & de fatima oliveira 2012 | 131 | Public sphere | Papacharissi, Z., & de Fatima Oliveira, M. (2012). Affective news and networked publics: The rhythms of news storytelling on #Egypt. *Journal of Communication, 62*(2), 266–282. |
| baer 2016 | 128 | Public sphere | Baer, H. (2016). Redoing feminism: Digital activism, body politics, and neoliberalism. *Feminist Media Studies, 16*(1), 17–34. |
| yang 2016 | 125 | Public sphere | Yang, G. (2016). Narrative agency in hashtag activism: The case of #BlackLivesMatter. *Media and Communication, 4*(4), 13–17.‏ |
| boyd 2010 | 120 | Public sphere | Boyd, D. (2010). Social network sites as networked publics: Affordances, dynamics, and implications. In Z. Papacharissi (Ed.), *A networked self* (pp. 47–66). Routledge. ‏ |
| clark 2016 | 120 | Public sphere | Clark, R. (2016). “Hope in a hashtag”: The discursive activism of #WhyIStayed. *Feminist Media Studies, 16*(5), 788–804. |
| mendes et al. 2018 | 113 | Public sphere | Mendes, K., Ringrose, J., & Keller, J. (2018). #MeToo and the promise and pitfalls of challenging rape culture through digital feminist activism. *European Journal of Women's Studies, 25*(2), 236–246..‏ |
| van dijck 2013 | 112 | Public sphere | Van Dijck, J. (2013). *The culture of connectivity: A critical history of social media*. Oxford University Press. |
| papacharissi 2015 | 110 | Public sphere | Papacharissi, Z. (2015). *Affective publics: Sentiment, technology, and politics.* Oxford University Press.‏ |
| freelon et al. 2016 | 99 | Public sphere | Freelon, D., McIlwain, C. D., & Clark, M. (2016). Beyond the hashtags: #Ferguson, #Blacklivesmatter, and the online struggle for offline justice. Center for Media & Social Impact, American University. Retrieved from <https://ssrn.com/abstract=2747066>.‏ |
| jackson et al. 2020 | 97 | Public sphere | Jackson, S. J., Bailey, M., & Welles, B. F. (2020). *#HashtagActivism: Networks of race and gender justice*. MIT Press.‏ |
| tremayne 2014 | 93 | Public sphere | Tremayne, M. (2014). Anatomy of protest in the digital era: A network analysis of Twitter and Occupy Wall Street. *Social Movement Studies, 13*(1), 110–126. |
| marwick & boyd 2011b | 91 | Public sphere | Marwick, A. E., & Boyd, D. (2011). I tweet honestly, I tweet passionately: Twitter users, context collapse, and the imagined audience. *New Media & Society, 13*(1), 114–133. |
| meraz & papacharissi 2013 | 87 | Public sphere | Meraz, S., & Papacharissi, Z. (2013). Networked gatekeeping and networked framing on #Egypt. *The International Journal of Press/Politics, 18*(2), 138–166. |
| blondel et al. 2008 | 86 | Public sphere | Blondel, V. D., Guillaume, J. L., Lambiotte, R., & Lefebvre, E. (2008). Fast unfolding of communities in large networks. *Journal of Statistical Mechanics: Theory and Experiment, 2008*(10), P10008..‏ |
| blei et al. 2003 | 85 | Public sphere | Blei, D. M., Ng, A. Y., & Jordan, M. I. (2003). Latent Dirichlet allocation. *Journal of Machine Learning Research, 3*(Jan), 993–1022. |
| youmans & york 2012 | 84 | Public sphere | Youmans, W. L., & York, J. C. (2012). Social media and the activist toolkit: User agreements, corporate interests, and the information infrastructure of modern social movements. *Journal of Communication, 62*(2), 315–329. |
| penney & dadas 2014 | 83 | Public sphere | Penney, J., & Dadas, C. (2014). (Re)Tweeting in the service of protest: Digital composition and circulation in the Occupy Wall Street movement. *New Media & Society, 16*(1), 74–90. |
| brock 2012 | 81 | Public sphere | Brock, A. (2012). From the blackhand side: Twitter as a cultural conversation. *Journal of Broadcasting & Electronic Media, 56*(4), 529–549. |
| bennett & segerberg 2012 | 797 | Collective action | Bennett, W. L., & Segerberg, A. (2012). The logic of connective action. *Information, Communication & Society*, *15*(5), 739–768.‏ |
| castells 2015 [2012] | 615 | Collective action | Castells, M. (2015 [2012]). *Networks of outrage and hope: Social movements in the Internet age*. John Wiley & Sons.‏ |
| gerbaudo 2012 | 380 | Collective action | Gerbaudo, P. (2012). *Tweets and the streets: Social media and contemporary activism*. Pluto Press.‏ |
| benford & snow 2000 | 262 | Collective action | Benford, R. D., & Snow, D. A. (2000). Framing processes and social movements: An overview and assessment. *Annual Review of Sociology, 26*(1), 611–639. |
| juris 2012 | 214 | Collective action | Juris, J. S. (2012). Reflections on #Occupy Everywhere: Social media, public space, and emerging logics of aggregation. *American Ethnologist, 39*(2), 259–279. |
| bennett & segerberg 2013 | 207 | Collective action | Bennett, W. L., & Segerberg, A. (2013). *The logic of connective action: Digital media and the personalization of contentious politics*. Cambridge University Press.‏ |
| mccarthy & zald 1977 | 165 | Collective action | McCarthy, J. D., & Zald, M. N. (1977). Resource mobilization and social movements: A partial theory. *American Journal of Sociology, 82*(6), 1212–1241. |
| bennett 2012 | 140 | Collective action | Bennett, W. L. (2012). The personalization of politics: Political identity, social media, and changing patterns of participation. *The Annals of the American Academy of Political and Social Science, 644*(1), 20–39. |
| entman 1993 | 139 | Collective action | Entman, R. M. (1993). Framing: Toward clarification of a fractured paradigm. *Journal of Communication, 43*(4), 51–58. |
| melucci 1996 | 132 | Collective action | Melucci, A. (1996). The process of collective identity. In *Challenging Codes: Collective Action in the Information Age* (pp. 68–86). Cambridge University Press. |
| gerbaudo & trerž 2015 | 129 | Collective action | Gerbaudo, P., & Treré, E. (2015). In search of the 'we' of social media activism: Introduction to the special issue on social media and protest identities. *Information, Communication & Society, 18*(8), 865–871. |
| kavada 2015 | 128 | Collective action | Kavada, A. (2015). Creating the collective: Social media, the Occupy Movement and its constitution as a collective actor. *Information, Communication & Society, 18*(8), 872–886. |
| bennett & segerberg 2011 | 125 | Collective action | Bennett, W. L., & Segerberg, A. (2011). Digital media and the personalization of collective action: Social technology and the organization of protests against the global economic crisis. *Information, Communication & Society, 14*(6), 770–799. |
| snow et al. 1986 | 119 | Collective action | Snow, D. A., Rochford Jr, E. B., Worden, S. K., & Benford, R. D. (1986). Frame alignment processes, micromobilization, and movement participation. *American Sociological Review, 51*, 464–481. |
| tilly 1978 | 113 | Collective action | Tilly, C. (1978). *From mobilisation to revolution*. Random House.‏ |
| della porta & diani 2020 [2006] | 108 | Collective action | della Porta, D., & Diani, M. (2020 [2006]). *Social Movements: An Introduction*. Blackwell. |
| goffman 1974 | 107 | Collective action | Goffman, E. (1974). *Frame analysis: An essay on the organization of experience.* Harvard University Press.‏ |
| milan 2015 | 106 | Collective action | Milan, S. (2015). From social movements to cloud protesting: The evolution of collective identity. *Information, Communication & Society, 18*(8), 887–900.‏ |
| snow & benford 1988 | 105 | Collective action | Snow, D. A., & Benford, R. D. (1988). Ideology, frame resonance, and participant mobilization. *International Social Movement Research, 1*(1), 197–217. |
| bennett et al. 2014 | 103 | Collective action | Bennett, W. L., Segerberg, A., & Walker, S. (2014). Organization in the crowd: Peer production in large–scale networked protests. *Information, Communication & Society, 17*(2), 232–260. |
| polletta & jasper 2001 | 98 | Collective action | Polletta, F., & Jasper, J. M. (2001). Collective identity and social movements. *Annual Review of Sociology, 27*(1), 283–305.‏ |
| bimber et al. 2012 | 94 | Collective action | Bimber, B., Flanagin, A., & Stohl, C. (2012). *Collective action in organizations: Interaction and engagement in an era of technological change*. Cambridge University Press. |
| olson jr 1965 | 82 | Collective action | Olson Jr, M. (1965). *The Logic of Collective Action: Public Goods and the Theory of Groups*. Harvard University Press. |
| gamson 1992 | 80 | Collective action | Gamson, W. A. (1992). *Talking Politics*. Cambridge University Press.‏ |
| earl & kimport 2011 | 303 | Communication and media | Earl, J., & Kimport, K. (2011). *Digitally Enabled Social Change: Activism in the Internet Age*. MIT Press.‏ |
| shirky 2008 | 223 | Communication and media | Shirky, C. (2008). *Here comes everybody: The power of organizing without organizations.* Penguin.‏ |
| segerberg & bennett 2011 | 208 | Communication and media | Segerberg, A., & Bennett, W. L. (2011). Social media and the organization of collective action: Using Twitter to explore the ecologies of two climate change protests. *The Communication Review, 14*(3), 197–215.‏ |
| cropf 2008 | 154 | Communication and media | Cropf, R. A. (2008). Review of Benkler, Y. (2006). *The Wealth of Networks: How Social Production Transforms Markets and Freedom*. New Haven and London: Yale University Press. 528 pp. $40.00 (papercloth). *Social Science Computer Review, 26*(2), 259–261.‏ |
| garrett 2006 | 150 | Communication and media | Garrett, R. K. (2006). Protest in an information society: A review of literature on social movements and new ICTs. *Information, Communication & Society, 9*(2), 202–224.‏ |
| chadwick 2013 | 144 | Communication and media | Chadwick, A. (2013). *The hybrid media system: Politics and power*. Oxford Studies in Digital Politics. |
| jenkins 2006 | 139 | Communication and media | Jenkins, H. (2006). *Convergence culture: Where old and new media collide*. New York University Press. |
| bimber et al. 2005 | 138 | Communication and media | Bimber, B., Flanagin, A. J., & Stohl, C. (2005). Reconceptualizing collective action in the contemporary media environment. *Communication Theory, 15*(4), 365–388. |
| harlow 2012 | 132 | Communication and media | Harlow, S. (2012). Social media and social movements: Facebook and an online Guatemalan justice movement that moved offline. *New Media & Society, 14*(2), 225–243. |
| van laer & van aelst 2010 | 131 | Communication and media | Van Laer, J., & Van Aelst, P. (2010). Internet and social movement action repertoires: Opportunities and limitations. *Information, Communication & Society, 13*(8), 1146–1171. |
| castells 2013 | 121 | Communication and media | Castells, M. (2013). *Communication power*. Oxford University Press.‏ |
| castells 2007 | 110 | Communication and media | Castells, M. (2007). Communication, power and counter-power in the network society. *International Journal of Communication, 1*(1), 29. |
| morozov 2011a | 104 | Communication and media | Morozov, E. (2011). *The net delusion: How not to liberate the world*. Penguin.‏ |
| gitlin 2003 | 102 | Communication and media | Gitlin, T. (2003). *The whole world is watching: Mass media in the making and unmaking of the new left*. University of California Press.‏ |
| gamson & wolfsfeld 1993 | 91 | Communication and media | Gamson, W. A., & Wolfsfeld, G. (1993). Movements and media as interacting systems. *The Annals of the American Academy of Political and Social Science, 528*(1), 114–125. |
| lievrouw 2011 | 89 | Communication and media | Lievrouw, L. A. (2011). *Alternative and activist new media*. Wiley.‏ |
| van de donk et al. 2004 | 86 | Communication and media | Van de Donk, W., Loader, B. D., Nixon, P. G., & Rucht, D. (Eds.). (2004). *Cyberprotest: New media, citizens and social movements*. Routledge.‏ |
| chadwick 2007 | 80 | Communication and media | Chadwick, A. (2007). Digital network repertoires and organizational hybridity. *Political Communication, 24*(3), 283–301. |

**Section E: Co-occurrence Keywords Data**

Table E1 shows the 100 most frequent keywords and their occurrences in each period of the periodic maps.

Table E1: Frequent keywords by period.

| **2010-2014** |  | **2015-2019** |  | **2020-2023** |  |
| --- | --- | --- | --- | --- | --- |
| social media | 265 | social media | 1234 | social media | 1573 |
| internet | 141 | twitter | 398 | twitter | 539 |
| media | 79 | internet | 341 | activism | 517 |
| twitter | 77 | social movements | 336 | protests | 428 |
| social movements | 76 | activism | 331 | politics | 343 |
| activism | 63 | protests | 304 | social movements | 330 |
| collective action | 61 | media | 293 | media | 323 |
| arab spring | 60 | politics | 243 | facebook | 258 |
| social networks | 55 | facebook | 231 | internet | 248 |
| communication | 54 | communication | 192 | communication | 222 |
| protests | 51 | movements | 192 | movements | 211 |
| facebook | 50 | participation | 185 | mobilization | 196 |
| politics | 39 | collective action | 178 | participation | 196 |
| participation | 38 | mobilization | 177 | collective action | 195 |
| networks | 37 | online | 165 | online | 179 |
| mobilization | 33 | information | 152 | identities | 178 |
| online | 32 | networks | 130 | covid-19 | 173 |
| movements | 29 | social networks | 127 | digital activism | 168 |
| information | 28 | identities | 106 | information | 155 |
| web 2.0 | 27 | news | 103 | news | 137 |
| revolution | 26 | political participation | 103 | gender | 132 |
| technologies | 26 | digital activism | 99 | engagement | 131 |
| egypt | 24 | technologies | 86 | networks | 126 |
| news | 24 | engagement | 84 | social networks | 111 |
| political participation | 23 | democracy | 76 | political participation | 110 |
| youtube | 22 | power | 70 | impact | 105 |
| democracy | 21 | gender | 69 | discourses | 103 |
| engagement | 20 | feminism | 67 | #blacklivesmatter | 101 |
| youth | 18 | arab spring | 65 | feminism | 98 |
| new media | 17 | civic engagement | 65 | power | 98 |
| digital media | 16 | digital media | 64 | youth | 90 |
| public sphere | 16 | organization | 63 | race | 88 |
| communities | 15 | race | 56 | instagram | 87 |
| models | 15 | youth | 56 | models | 87 |
|  |  | communities | 55 | hashtags | 86 |
|  |  | political communication | 52 | emotions | 84 |
|  |  | violence | 52 | violence | 83 |
|  |  | youtube | 51 | democracy | 81 |
|  |  | china | 50 | women | 81 |
|  |  | elections | 50 | china | 79 |
|  |  | impact | 50 | #metoo | 77 |
|  |  | public sphere | 50 | culture | 75 |
|  |  | behavior | 48 | populism | 75 |
|  |  | connective action | 45 | climate change | 73 |
|  |  | emotions | 45 | strategies | 73 |
|  |  | models | 45 | hashtag activism | 72 |
|  |  | framing | 43 | communities | 71 |
|  |  | identities | 42 | digital media | 69 |
|  |  | discourses | 42 | technologies | 67 |
|  |  | news coverage | 41 | political communication | 66 |
|  |  | revolution | 41 | resistance | 62 |
|  |  | women | 41 | civic engagement | 60 |
|  |  | online activism | 40 | behavior | 58 |
|  |  | journalism | 39 | connective action | 56 |
|  |  | united states | 39 | framing | 55 |
|  |  | egypt | 38 | news coverage | 55 |
|  |  | knowledge | 38 | perceptions | 55 |
|  |  | big data | 37 | attitudes | 54 |
|  |  | organizations | 37 | memes | 53 |
|  |  | censorship | 36 | health | 52 |
|  |  | dynamics | 36 | elections | 51 |
|  |  | culture | 34 | online activism | 51 |
|  |  | occupy wall street | 34 | social network analysis | 51 |
|  |  | age | 33 | youtube | 50 |
|  |  | campaigns | 32 | management | 49 |
|  |  | content analysis | 32 | dynamics | 48 |
|  |  | space | 32 | sentiment analysis | 47 |
|  |  | civil society | 31 | intersectionality | 46 |
|  |  | perceptions | 30 | hong kong | 44 |
|  |  | resistance | 30 | justice | 44 |
|  |  | social network analysis | 30 | organizations | 44 |
|  |  | citizenship | 29 | images | 42 |
|  |  | education | 29 | racism | 42 |
|  |  | hashtags | 29 | knowledge | 41 |
|  |  | icts | 29 | conflict | 40 |
|  |  | surveillance | 29 | polarization | 39 |
|  |  | advocacy | 28 | public sphere | 39 |
|  |  | expression | 28 | campaigns | 38 |
|  |  | collective identity | 27 | censorship | 38 |
|  |  | policy | 27 | collective identity | 38 |
|  |  | populism | 27 | crisis | 38 |
|  |  | strategies | 27 | journalism | 38 |
|  |  | turkey | 27 | policy | 38 |
|  |  | health | 26 | big data | 37 |
|  |  | sites | 26 | content analysis | 37 |
|  |  | diffusion | 25 | people | 37 |
|  |  | hashtag activism | 25 | affordances | 36 |
|  |  | new media | 25 | fake news | 36 |
|  |  | public opinion | 25 | framework | 36 |
|  |  | attitudes | 24 | machine learning | 36 |
|  |  | patterns | 24 | misinformation | 36 |
|  |  | trust | 24 | performance | 36 |
|  |  | exposure | 23 | public opinion | 36 |
|  |  | memes | 23 | sexual violence | 35 |
|  |  | online communities | 23 | civil society | 34 |
|  |  | opinion | 23 | surveillance | 34 |
|  |  | political protest | 23 | trust | 34 |
|  |  | affect | 22 | disinformation | 33 |
|  |  | climate change | 22 | experiences | 33 |
|  |  | conflict | 22 | exposure | 33 |

**Section F: Computational Historical Analysis of Methods**

Table F1 shows the methodological categories, their respective search terms, and the number of results for each category in the dataset. The search terms were adapted from Walter and Ophir (2024), and were applied to the abstract, keywords, and keywords plus fields using analytical code in R that is available in the article’s replication files.

Table F1: Search terms for methods evolution analysis.

| **Methods category** | **Search terms** | **Results in Dataset** |
| --- | --- | --- |
| Qualitative | "focus group", "interview", "semi-structured", "in-depth interview", "group discussion", "qualitative interview", "qualitative research", "dense description" | 811 |
| Surveys | “survey”, “cross-section” (with optional hyphenation), "longitudinal survey", "panel survey", "wave survey", "repeated survey", "time-series survey" | 571 |
| Content analysis | "content analysis" (excluding automated/computer-assisted/computerized variations), "text analysis" (excluding automated variations), "manual content analysis", "human-coded" | 560 |
| Computational content analysis | "topic modeling", "automated image", "data mining", "automated content/text analysis", "computer-assisted content/text analysis", "computerized content/text analysis", "machine learning", "natural language processing", "nlp", "sentiment analysis", "text mining", "automated coding", "dictionary-based analysis", "computational linguistics", "computer-aided text analysis", "supervised learning", "unsupervised learning", "text as data", "images as data" | 381 |
| Ethnography | "ethnograph" (captures ethnography/ethnographic), "participant observation", "participant research", "fieldwork" (excluding quantitative fieldwork), "field-work" (hyphenated variation), "digital ethnography" | 328 |
| Network analysis | "network analysis", "semantic net", "social network analysis", "citation network", "co-citation", "network visualization", "network metrics", "network centrality", "network structure" | 283 |
| Discourse analysis | "discourse analysis" (with optional hyphenation) | 215 |
| Mixed methods | "mixed method", "multi method" (also capturing hyphenation and use of plural), "triangulation/triangulated", "qualitative comparative analysis", "QCA", "fsQCA/csQCA/mvQCA" (variants), "process tracing", "sequential explanatory/exploratory design/approach", "convergent parallel/design/approach", "embedded design", "case-based analysis" | 200 |
| Comparative | "comparative case/analysis/study/research/method", "cross-national comparison", "cross-cultural comparison", "longitudinal comparison" (excluding survey-based) | 78 |
| Systematic reviews | "systematic lit" (also systematic literature), "systematic review", "meta-analysis", "metaanalysis" (unhyphenated variation) | 64 |
| Experiments | "experimental condition/group/treatment/manipulation/stimuli/between-subjects/within-subjects", "random assign" (with flexible spacing/hyphenation), "controlled experiment", "between-subjects design", "within-subjects design", "factorial design", "participants/subjects were randomly assigned”, "vignette study/experiment/design" | 17 |

**References for Supplemental Materials**

Burgess, J., Marwick, A., & Poell, T. (Eds.). (2018). *The Sage handbook of social media*. Sage.

Della Porta, D., & Diani, M. (Eds.). (2015). *The Oxford handbook of social movements*. Oxford University Press.

Huang, Y., Schuehle, J., Porter, A. L., & Youtie, J. (2015). A systematic method to create search strategies for emerging technologies based on the Web of Science: Illustrated for ‘Big Data.’ *Scientometrics*, *105*(3), 2005–2022. <https://doi.org/10.1007/s11192-015-1638-y>

van Eck, N. J., & Waltman, L. (2023). *Manual for VOSviewer version 1.6.20*. Univeristeit Leiden. <https://www.vosviewer.com/documentation/Manual_VOSviewer_1.6.20.pdf>

Walter, D., & Ophir, Y. (2024). Meta-theorizing framing in communication research (1992–2022): Toward academic silos or professionalized specialization? *Journal of Communication*, *74*(2), 101–116. <https://doi.org/10.1093/joc/jqad043>
